# Potassium Uptake Systems in *Staphylococcus aureus*: New Stories about Ancient Systems

**DOI:** 10.1128/mBio.00784-13

**Published:** 2013-10-08

**Authors:** Angelika Gründling

**Affiliations:** Section of Microbiology and MRC Centre for Molecular Bacteriology and Infection, Imperial College London, London, United Kingdom

## Abstract

*Staphylococcus aureus* is a hardy organism that can survive high-salt conditions better than many other bacteria. This characteristic is thought to help *S. aureus* survive in the nares and on the skin of the human host and is used to selectively propagate and identify *Staphylococcus* species. However, the mechanism that allows *S. aureus* to tolerate such high-salt conditions is not well understood. A recent study in *mBio* by A. Price-Whelan et al. [mBio 4(4):e00407-13, 2013, doi:10.1128/mBio.00407-13] highlights the importance of potassium uptake in this process. This commentary provides a perspective of the study by Price-Whelan et al. as well as other recently reported work on potassium uptake and transport systems in *S. aureus*.

## Commentary

Potassium is the major monovalent intracellular cation in cells, and its uptake is essential for all living organisms. It has many key functions within bacterial cells: potassium is required for the activity of intracellular enzymes, acts as an intracellular second messenger, and is involved in the maintenance of a constant internal pH and membrane potential (for a review, see reference 1). In addition, potassium plays an important function as an osmotic solute. In many bacteria, an increase in the intracellular potassium concentration is seen as a response to increases in the external NaCl concentration. The importance of potassium in cellular homeostasis is also revealed by the finding that bacteria usually express multiple specific uptake systems, which allow these organisms to maintain high intracellular potassium concentrations against concentration gradients. In general, bacteria are capable of accumulating intracellular potassium at concentrations 100 times greater than that in the surrounding solution, and *Staphylococcus aureus* maintains a high intracellular concentration of 0.5 to 1.5 M even if the extracellular concentration is in the low millimolar range (2, 3).

Potassium transport systems are ancient systems found among organisms from all three kingdoms of life, and they are likely derived from potassium channels (1, 4, 5). For bacteria, four different types of potassium transport systems have been described to date (1). Much of the pioneering work on potassium uptake has been performed in *Escherichia coli*, which expresses three transporters, Trk, Kdp, and Kup (1). Kdp is a high-affinity multicomponent transporter in which potassium uptake is powered by ATP hydrolysis and a protein phosphorylation-dephosphorylation cycle. In *E. coli*, Kdp expression is induced at a very low external potassium concentration (usually in the micromolar range) or at high osmolarity. The Kdp transporter has also been found in *S. aureus* (6, 7); however, until the recent studies by Price-Whelan et al. (8) and Gries et al. (9), it was thought to be nonfunctional. A fourth bacterial potassium transporter, designated Ktr, is absent from *E. coli* but is otherwise found in a wide variety of microbes, including *S. aureus*.

**FIG 1:**
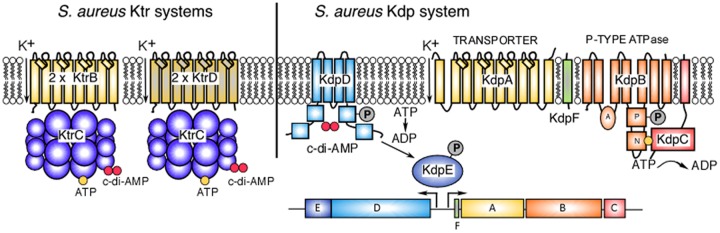
Potassium uptake systems in *S. aureus*. *S. aureus* has two different types of potassium uptake systems. The constitutively expressed Ktr system, shown on the left, is composed of the dimeric membrane components KtrB and KtrD and the octameric cytoplasmic gating component KtrC. Transporter activity is regulated by nucleotide binding to the cytoplasmic gating component, where ATP binding increases transporter activity (the effect on transporter activity upon c-di-AMP binding to KtrC has not yet been determined). The second potassium uptake system is the inducible Kdp system, shown on the right. Its expression is controlled by the two-component system KdpDE. The sensor histidine kinase KdpD is another c-di-AMP receptor protein, and it functions together with the transcription factor KdpE to activate the expression of the transporter components KdpFABC. The Kdp proteins show high homology to the corresponding *E. coli* proteins, and it can be assumed that KdpA is the membrane component, which makes up the potassium conduit (20). The energy for the potassium transport is proved by KdpB, a P-type ATPase, which binds and hydrolyzes ATP and undergoes a phosphorylation and dephosphorylation cycle (20). KdpC is embedded in the membrane via an N-terminal transmembrane helix and is thought to increase the affinity of KtrB for ATP. Last, KdpF, a small hydrophobic protein with a single transmembrane domain, has been suggested to provide stability to the Kdp complex.

Unlike many other microbes, *S. aureus* maintains a high intracellular potassium concentration even in the absence of osmotic stress, and osmotic stress does not necessarily increase its concentration (2). The work performed by Price-Whelan et al. and two other recent studies revealed that potassium uptake systems are nevertheless essential for *S. aureus* to cope with osmotic stress caused by NaCl (8–10). Based on these recent observations, the following views have emerged: *S. aureus* contains two types of specific potassium uptake systems, Ktr and Kdp (Fig. 1). Even when both systems are deleted, *S. aureus* mutants continue to grow provided sufficiently high concentrations of potassium are present in the growth medium, indicating that potassium enters staphylococci through alternative routes, as has been reported for *E. coli* (9, 11).

The constitutively expressed Ktr system is a little different in *S. aureus* than in other bacteria (8, 9). This system, which consists of a dimeric membrane and an octameric cytoplasmic gating component, was first characterized for the Gram-positive bacterium *Enterococcus hirae* and for the Gram-negative bacterium *Vibrio alginolyticus* (12, 13). *Bacillus subtilis* harbors two Ktr transporters: KtrAB, which is encoded within a single operon, and KtrCD, the components of which are encoded at different positions on the chromosome (14). In *S. aureus*, the system is composed of the dimeric membrane components KtrB and KtrD but only a single octameric cytoplasmic gating component, which shows greater homology to *B. subtilis* KtrC than to the KtrA protein (8, 9). While in *B. subtilis* the respective membrane and gating components can only function together, recent work by Gries et al. indicated that in *S. aureus* KtrC can form an active potassium transporter with either one of the membrane components (9). An *S. aureus ktrC* mutant or a *ktrB ktrD* double mutant (but not the single mutants alone) displayed growth defects under potassium-limiting condition in chemically defined medium (9). Furthermore, all three recent publications on the *S. aureus* Ktr system showed that *ktrC* mutants display a growth defect in high-osmolarity medium, particularly when the potassium concentration is limiting (8–10).

It is thought that K^+^ transport through Ktr systems requires the cotransport of Na^+^ ions and that the potassium flux through the membrane component is further controlled by the cytoplasmic gating component and its interaction with nucleotides (but not by hydrolysis of nucleotides) (15, 16). An exciting new development was the recent determination of the structure of the complete *B. subtilis* KtrAB transporter (17). This study provided important information on the molecular interaction between the dimeric KtrB membrane component and the octameric KtrA gating ring and showed that transport activity is increased upon ATP binding to the gating component. The authors also proposed an alternative model for the gating mechanism of Ktr transporters that involves the binding of a second ligand for full activation of the transporter (17). Serendipitously, studies of the signaling nucleotide c-di-AMP have revealed that c-di-AMP can also interact with the cytoplasmic gating component of Ktr systems (10). The two nucleotides interact at different sites; ATP binds within the N-terminal domain of the KtrA/C protein, whereas c-di-AMP interacts with the C-terminal domain (10). However, whether c-di-AMP is indeed the second activating ligand, as proposed by Vieira-Pires et al., still requires further investigation (17).

Several c-di-AMP receptor proteins have now been identified (recently reviewed in reference 18), and it is interesting to note that another c-di-AMP binding protein is KdpD (10). As shown in the study by Price-Whelan et al., this protein is the two-component system histidine kinase that functions, together with the transcriptional factor KdpE, to induce the expression of the high-affinity potassium uptake system KdpFABC. The *kdp* genes are highly induced in complex medium under high-osmolarity conditions caused by the addition of NaCl or sucrose but not KCl (8). For *E. coli*, it has recently been proposed that direct binding of potassium to KdpD prevents activation of the system (19), and the existence of a similar mechanism in *S. aureus* could explain its lack of responsiveness at high potassium concentrations. It should be noted, however, that a *S. aureus* mutant lacking a functional Kdp uptake system did not show a significant growth defect when propagated in complex medium containing a high NaCl concentration. Hence, under these conditions, the Ktr and/or unspecific uptake systems are sufficient to supply bacterial cells with potassium (8). However, a *kdpA* mutant was unable to grow in chemically defined medium with an extremely low potassium concentration of 10 µM (8), an observation with important implications, since it establishes that the Kdp system is indeed a functional potassium uptake system in *S. aureus*, a matter that was questioned in previous studies (6). This, in turn, suggests that the signaling nucleotide c-di-AMP is involved in the control of the two main potassium uptake systems in *S. aureus* and likely also several other Gram-positive bacteria.

We now have an overview of potassium transport activity in *S. aureus*, and recent studies have established the Ktr and Kdp systems as the two main active systems in this organism. Nevertheless, significant differences in the severity of growth defects and phenotypes were observed even when seemingly similar *S. aureus* strains were analyzed. These observations underscore the importance of careful experimental control of osmolarity and pH in growth media in addition to determining the residual potassium concentration, even in chemically defined medium, when studying potassium transport. Recent work has also highlighted the unexpected complexity of the gating mechanism and control of potassium transport, and the molecular details of how c-di-AMP signaling impacts this pathway are only beginning to emerge.
